# A Country Doctor’s First Case of Surgery

**Published:** 1887-08

**Authors:** R. H. Day

**Affiliations:** Baton Rouge, La., Ex-President Louisiana State Medical Association


### A COUNTRY DOCTOR'S FIRST CASE OF SURGERY.

*By R. H. Day, M. D., Baton Rouge, La., Ex-President Louisiana State Medical Association.*

For Daniel's Texas Medical Journal.

COUNTRY DOCTORS, generally esteemed as occupying an inferior position among the educated city fraternity, are nevertheless an important integral part of the medical profession; and, although their medical training and hospital advantages are greatly superior now to what they were a half century ago, still they must yet necessarily commence their professional career lacking that solid experience which inspires confidence in the hour of danger and grave emergencies.

Whatever there can be gleaned from the experience of one who has passed half a century amid the labors and responsibilities of a country medical practice, and rescue from oblivion the facts and incidents that will instruct and encourage the undervalued country doctor, and that will add something of value to the general history of medicine, should be acceptable, as it must be useful to all classes of physicians.

Impelled by an honorable consideration for my future professional success in life, in the early part of the year 1837, I left my native State (Maryland), where society was fixed and stable, and every household had its regular and abiding family doctor, leaving but slender and fortuitous opportunities for the young graduate, however well prepared, ever to succeed in practice and rise to distinction in his profession, and cast my lot in the young and growing State of Illinois, and in the beautiful little town of Mt. Carmel. Here society was mixed, constituted of people from the different States of the Union and of the several nations of Europe, and constantly added to by fresh immigrants from these same sources.

In this new field, professional competition was open and more equal, and occasions frequently transpired to develop talent and to test the mental strength and qualifications of the disciples of Esculapius.

The narration of the following case, with its incidents, will serve to illustrate and exemplify their nature, as also the trying responsibilities connected therewith:

My first case of surgery presented itself in 1839, being requested by Dr. A., living some ten or twelve miles distant, to perform an amputation for him. It was a thigh amputation, and being my first attempt, never before having ever amputated even a toe or a finger, I may be pardoned for relating, briefly, some of the incidents, and my own feelings on that thrilling occasion.

First, then, the subject was a young (white) female, very delicate and greatly emaciated from an extensive chronic, scrofulous ulceration of the left leg, and well marked pulmonary tuberculosis; liable on that account to make the operation a failure—and the possibility, and even probability, that she might die while undergoing the operation. Secondly, the operation had to be done at the patient's home, twenty miles distant in the country, with no conveniences, except of the most primitive character, such as could be improvised under the unfavorable surroundings of the locality, and with the assistance only of the attending physician (who, by the way, was a most stirling man and a skillful practitioner of medicine, but with only the anatomical advantages which had been derived by attendance upon one course of lectures in the University of Pennsylvania), together with a few neighbors, drawn hither by curiosity to witness what they had never before seen.

Under such conditions, with the performance of what was then, and is still, regarded as a capital operation, a young country doctor, without any practical experience, was to make his debut on the field of operative surgery. My feelings can well be imagined by those of the profession who have been subjected to a similar ordeal.

Standing beside my pale emaciated patient, I realized my awful responsibility, and felt it to be a moment of supreme effort, my fears and resolutions alternately in the supremacy. Fortunately, when attending medical lectures, I had given special and close attention to anatomy and dissections, and I was conscious I could rely upon my familiarity with the anatomy and relation of the parts to each other, as well as the rules laid down by the masters in surgery for the performance of such operation ; but in despite of me, I would become tremulous and almost giddy when my mind pictured the cleaving knife, the quivering muscle and the streaming blood of the poor exhausted female before me,—and perhaps to die from shock, exhaustion or hemorrhage under the operation. I turned quickly about to banish my timid thoughts and regain my waning manhood. I must and will be equal to the demands of this occasion, or forfeit my self-respect and all aspirations or hope of future success in my chosen profession. Advancing to my patient's side with a firm step, I suggested that she should take some whiskey and laudanum with the view of stimulating and bracing her up to bear the operation; but she resolutely refused, saying, she knew that the pain of the operation could not equal the suffering which she had borne for months past, and that she had prepared her mind to see and bear it all.

Such cool and determined courage inspired me with hope and confidence, and the arrangements and prepartions being ready, she was slipped down over the foot of the bed upon which she was lying, and yielding to her wishes, her head and shoulders were well elevated, her legs resting upon two chairs supported by assistants, I at once proceeded to amputate by the circular method, taking off the limb very high up to avoid the diseased tissues.

I omit details, not to be tedious. Let it suffice to say, that the operation was completed *secundum artem* without a mishap or a balk, the arteries ligated, and the stump dressed. It was a success —a country doctor had triumphed over his fears, and achieved a success under the most unfavorable and unpropitious conditions, of which, even our learned and distinguished city surgeons, might well be proud.

The patient made a good recovery from the operation, and for several months her general health apparently improved; she gaining in flesh and spirits. The following winter, however, her lung trouble became aggravated, from which she died about twelve months subsequent to the operation.

This case is now reported, not as possessing any particular merit in and of the operation, *per se*, since such operations are not of infrequent occurrence, but is given as a contribution to the surgical history of this century, collected from the experience of an obscure country doctor; important and instructive, as his first effort in surgery, and on account of the discouraging and unpropitious conditions attending it, and its favorable termination notwithstanding; and secondly, to encourage country doctors to cultivate independence of thought and self-reliance in their manhood; but above all to impress upon their minds the inexorable necessity and absolute duty of being thoroughly prepared and grounded in the knowledge of anatomy and the fundamental principles of general medicine, that they may win a proud success, and by their work and lives honor the noble profession that so munificently honors them.

